# Latin American immigrants have limited access to health insurance in Japan: a cross sectional study

**DOI:** 10.1186/1471-2458-12-238

**Published:** 2012-03-25

**Authors:** S Pilar Suguimoto, Masako Ono-Kihara, Mitchell D Feldman, Masahiro Kihara

**Affiliations:** 1 Department of Global Health and Socio-epidemiology, Kyoto University School of Public Health, Yoshida-Konoe-cho, Sakyo-ku, Kyoto 606-8501, Japan; 2 Department of Medicine, University of California San Francisco, 1545 Divisadero, Suite 315, San Francisco, CA 94143-0320, USA

**Keywords:** Health insurance, Immigrants, Foreign workers, Japan, Latin America

## Abstract

**Background:**

Japan provides universal health insurance to all legal residents. Prior research has suggested that immigrants to Japan disproportionately lack health insurance coverage, but no prior study has used rigorous methodology to examine this issue among Latin American immigrants in Japan. The aim of our study, therefore, was to assess the pattern of health insurance coverage and predictors of uninsurance among documented Latin American immigrants in Japan.

**Methods:**

We used a cross sectional, mixed method approach using a probability proportional to estimated size sampling procedure. Of 1052 eligible Latin American residents mapped through extensive fieldwork in selected clusters, 400 immigrant residents living in Nagahama City, Japan were randomly selected for our study. Data were collected through face-to-face interviews using a structured questionnaire developed from qualitative interviews.

**Results:**

Our response rate was 70.5% (n = 282). Respondents were mainly from Brazil (69.9%), under 40 years of age (64.5%) and had lived in Japan for 9.45 years (SE 0.44; median, 8.00). We found a high prevalence of uninsurance (19.8%) among our sample compared with the estimated national average of 1.3% in the general population. Among the insured full time workers (n = 209), 55.5% were not covered by the Employee's Health Insurance. Many immigrants cited financial trade-offs as the main reasons for uninsurance. Lacking of knowledge that health insurance is mandatory in Japan, not having a chronic disease, and having one or no children were strong predictors of uninsurance.

**Conclusions:**

Lack of health insurance for immigrants in Japan is a serious concern for this population as well as for the Japanese health care system. Appropriate measures should be taken to facilitate access to health insurance for this vulnerable population.

## Background

Japan provides universal health insurance and all citizens, including foreigners who stay for a year or more are required to enroll in one of the public health insurance schemes [1,2]. Health insurance coverage remains one of the most important ways to ensure access to health services [3]. With insurance, individuals and families can protect themselves against exceptional health care costs, and access screening services for early detection and treatment of illness [4,5]. Immigrants are a vulnerable population and their access to insurance and healthcare has been extensively researched in other countries [6-8]. Even in countries with universal health insurance such as Canada or Spain, immigrants face multiple barriers accessing healthcare [9,10], including uninsurance [11].

In Japan, foreigners represent 1.71% of the total population, roughly 2.2 million (as of 2009) [12]. Historically, immigration has played a minor role in Japan's history but the rapid growth of the Japanese economy in the 1980's coupled with a declining birth rate, aging population and younger Japanese labor force favoring higher status jobs, created a strong demand for labor in certain sectors of the economy [13,14]. The revision of the Immigration Law in 1990 led to a large influx of descendants of Japanese emigrants from Brazil and Peru. Brazilians alone are the third largest minority group (267, 500; 12.23%) after Chinese (680, 500; 31.13%) and Koreans (578, 500; 26.46%), but the occupational structure of Latin Americans differs from these groups. Latin Americans are overwhelmingly manual laborers in the manufacturing industry. In contrast, the occupations of other immigrant groups are more evenly spread between manual production workers, professional and technical, and personal services [15,16]. While in the early years of immigration Latin Americans were mostly temporary guest workers who intended to return home in a few years, more immigrants are settling in with their families [14].

Few prior studies have addressed the problems of healthcare access and uninsurance among foreign immigrants in Japan [17,18]. Findings from these studies are inconclusive because of the non-probabilistic nature of their sampling methods and the low response rates. Some authors speculate that uninsurance may be due in part to a reasoned decision by the immigrants themselves and also to a failure by many companies, especially labor contract companies, to provide insurance [14]. Our study was designed to assess the pattern of health insurance coverage and predictors of uninsurance among documented Latin Americans in Japan in a probability population sample.

## Methods

### Study setting

The study area, Nagahama City, lies on the eastern shore of Lake Biwa, the largest lake located close to the center of the Japan Peninsula. It extends 164.40 km^2^, divided into 298 townships with nearly 126,000 inhabitants [19]. This city hosts the largest number of Latin American immigrants in the west half of Japan because it is a major industrial center, where a number of automobile related companies employ foreign workers. In Nagahama City, nearly 2% of the population is Latin American [20], while they account for 0.3% of the total population in Japan [12].

### Survey instrument

We conducted a preliminary qualitative study in September of 2009. This initial step served several aims. It informed the development of an appropriate instrument for the quantitative survey and provided insight into recruitment issues [21,22]. Twenty Latin American immigrants (not included in the main survey) were recruited purposively in Nagahama and surrounding areas. After explaining the purpose of the study and obtaining individual consent we conducted semi-structured interviews, which lasted between 60 to 90 minutes, and upon completion provided a pre-paid monetary incentive (approximately 25.60 USD). Extensive notes were taken, as recording was not possible. Interviews focused on a range of topics including: current and past health insurance coverage; barriers for enrollment and payment; health status and access to medical care; work and accidents or problems related to the workplace. We also explored opinions, reasons and circumstances in known cases of uninsurance or personal cases of uninsurance. These findings were used to draft our survey instrument. We developed new questions, modified others found in the literature review [17], and also created response choices for domains related to work, health insurance, and health background and access to healthcare. The instrument was then assessed for its reliability in a test retest design with one week interval in 15 respondents, who were living in Nagahama but were not included in the final survey. All continuous variables showed a high test-retest reproducibility with Pearson correlation coefficient ranging 0.68 - 1.00. In general, categorical variables showed good agreement [23], except for some variables with kappa coefficients less than 0.60 that were excluded from the questionnaire.

The final survey instrument consisted of five domains: sociodemographic characteristics (12 items), work related (9 items), health insurance (19 items), health background and access to healthcare (14 items), and knowledge and opinion towards HIV/AIDS and HIV testing (14 items). The questions of the last domain were taken from the widely used guidelines for Behavioral Surveillance Surveys (BSS) [24]. Results from this domain, however, were not included in this paper. The instrument was translated into Spanish and Brazilian Portuguese by a bilingual researcher (SPS) and pilot tested for face validity.

### Sampling frame and sample selection

The target population was documented residents of Nagahama City from any Spanish or Portuguese speaking country in Latin America, aged 18 or over. Tourists and those who had overstayed their visa were excluded. Since all foreigners legally staying in Japan are registered in the township where they live and these statistics are readily available by township, we decided to use this information to create clusters. First we selected townships with more than 5 registered foreigners as of August 2010 [25] and when necessary we grouped neighboring townships to create 28 clusters having at least 30 immigrants. Then, 400 samples were selected according to a two-stage cluster sampling procedure. In the first stage, 25 clusters were selected with probability proportional to estimated size (PPES) with replacement [26], while in the second stage, 16 participants were randomly selected from each selected cluster without replacement. No information about age or nationality of registered foreigners in each township was available, but we assumed that it highly correlated with that of adult Latin Americans and used it as an estimated cluster size because nearly 80% of the foreign population in Nagahama was either Brazilian or Peruvian [27]. After mapping, this assumption was proven to be true because the correlation between the number of registered foreigners used as an estimated size of the clusters and the number of eligible Latin Americans identified in our field mapping in each township was very high (r = 0.98, *P *< 0.001).

**Figure 1:**
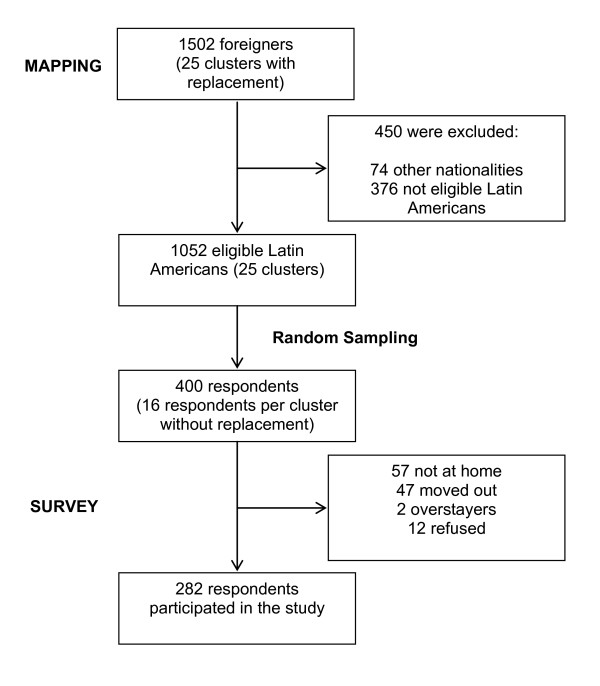
**Flow diagram of sampling procedures for Latin American immigrants in Nagahama City, Japan**.

Since an accurate address was not available for the foreign residents, we had to map the selected clusters to create the sampling frame. To get an idea of their residence distribution, we first collected information through neighborhood associations, private day care centers with foreign children, Japanese language class groups, a Brazilian school, religious associations and several key informants, and then started household visits. During November 2010 through March 2011, we visited initial contacts, labor contract companies' housings, public housings, and interviewed local inhabitants to confirm the presence of Latin American immigrants and collected basic information (gender, nationality, adult/minor and address). When necessary we visited the same dwellings several times, until we reached information saturation. During the mapping we collected information, directly or indirectly, on 1502 foreigners including nationalities other than Latin American such as Chinese, Korean, Philippine, Vietnamese, American, and Australian. Of these, 1052 Latin Americans were identified to be eligible and coded to generate an anonymous list as a frame for random sampling (Figure 1).

### Data Collection

The survey was conducted from March through May 2011. Data were collected through face-to-face interviews using a structured questionnaire. The interview was carried out by the lead author (SPS) and the fieldwork team in either Spanish or Brazilian Portuguese and took an average of 15 to 20 minutes to complete. Respondents who were not at home were visited up to 5 times on different hours and days of the week. All interviewers were trained on how to conduct the structured interview, informed of the study purpose, and closely supervised in the field. Interviews were conducted at a place designated by the participants. Before each interview the interviewer explained the purpose of the study, content of the questionnaire, and assured anonymity as well as confidentiality to each participant.

We considered Latin Americans to be those who were born and/or self identified as foreigners coming from Mexico, Central America (except Belize), Cuba, Dominican Republic, and South America (except Suriname, Guyana and French Guiana). Immigration status was addressed by asking the respondents what type of visa they had and if their visa was currently valid. Respondents were classified as uninsured if they reported not having any health insurance at the time of the interview, irrespective if they were the primary holder or dependant. Also, we asked if they knew about the mandatory nature of health insurance in Japan as it may not be the case in their countries of origin

### Analysis

Statistical analyses were performed using the Complex Samples module of SPSS version 17.0, a statistical analysis program that accounts for clustering and multiple stages of sampling [28]. All analyses incorporated appropriate standardized sample weights to adjust for nonresponse. Univariate analysis was used to describe the population and bivariate analysis to determine associations between uninsurance and other variables. Finally, factors significantly associated (p < 0.05) with being uninsured in the bivariate analyses were compulsorily entered into a multivariate logistic regression model to calculate adjusted odds ratios (AORs) to assess the magnitude of independent association of these predictors with uninsurance.

### Ethical issues

The study protocol was reviewed and approved by the Kyoto University Graduate School and Faculty of Medicine Ethics Committee. Verbal informed consent was obtained from all participants.

## Results

Of the 400 Latin American immigrants who were selected for the study, 57 (14.3%) were not at home, 47 (11.8%) had moved out, 2 (0.5%) were excluded from our study because they overstayed their visas and 12 refused to participate. Overall, 282 participants were included in our study (response rate 70.5%). All results that follow reflect weighted data. Table 1 displays the detailed characteristics of respondents. They consisted of slightly more women (51.5%), mostly from Brazil (69.9%), under 40 years of age (64.5%), married (70.4%), with children (75.4%), and reported 11.68 (SE 0.17; median, 12.00) years of formal education. Despite living 9.45 (SE 0.44; median, 8.00) years in Japan and 6.08 (SE 0.47; median, 5.00) years in Nagahama, only 29.2% reported having intentions to stay permanently in Japan (for more information see Additional file 1: Table S1.pdf)

**Table 1:** Sociodemographic, medical background and work related characteristics of documented Latin American immigrants in Nagahama City, Japan by health insurance status

	**Total**		**Uninsured**		**Insured**		***P *value**
	**(n = 282)**		**(n = 56)**		**(n = 226)**		
		
	**n**	**%^a^**	**n**	**%^a^**	**n**	**%^a^**	
**Sociodemographic**							
Gender							
Men	137	48.5	37	66.1	100	44.1	0.006
Women	145	51.5	19	33.9	126	55.9	
Country of origin							
Brazil	197	69.9	41	73.1	156	69.2	0.073
Peru	46	16.4	4	6.5	43	18.8	
Bolivia	25	8.9	10	18.2	15	6.6	
Others	14	4.8	1	2.2	12	5.4	
Age							
18 - 29	91	32.1	22	39.2	69	30.3	0.102
30 - 39	91	32.4	15	26.2	77	34.0	
40 - 49	55	19.4	16	28.1	39	17.3	
50 or more	45	16.0	4	6.5	42	18.4	
Marriage and live-in partnerships							
Married (living with spouse)	178	63.1	27	49.2	150	66.5	0.132
Married (not living with spouse or other partner)	20	7.3	3	6.0	17	7.6	
Not married (living with partner)	23	8.0	6	10.7	17	7.3	
Not married (not living with partner)	61	21.7	19	34.0	42	18.7	
Number of children							
0	69	24.6	26	45.8	44	19.3	0.045
1	80	28.5	16	28.0	65	28.6	
2	87	30.9	10	17.5	77	34.2	
3 or more	45	16.0	5	8.7	40	17.8	
Years of education							
1 - 8	41	14.5	16	29.1	25	10.9	0.013
9 -12	157	55.6	31	56.2	125	55.4	
13 -16	84	29.9	8	14.7	76	33.7	
**Knowledge about mandatory nature of health insurance in Japan**							
No	55	19.6	24	43.2	31	13.8	< 0.001
Yes	227	80.4	32	56.8	195	86.2	
**Medical background**							
Visited a doctor in the last 12 months in Japan^b^							
No/Have never visited a doctor in Japan	86	30.9	34	60.5	53	23.5	< 0.001
Yes	194	69.1	22	39.5	172	76.5	
Chronic disease^b^							
No	238	84.9	54	96.6	184	82.0	0.016
Yes	42	15.1	2	3.4	40	18.0	
**Work related**							
Working condition							
Full time	209	74.1	43	76.5	166	73.4	0.447
Part time	16	5.7	1	1.8	15	6.7	
Self employed	3	1.1	0	0.0	3	1.4	
Unemployed	53	18.6	12	21.6	40	17.9	
Retired	1	0.5	0	0.0	1	0.6	
Employed through labor contract company^c^							
No	58	25.7	11	24.8	47	27.1	0.852
Yes	167	74.3	33	75.2	134	72.9	
Years employed by the current employer^c^							
0 - 2	131	58.1	34	77.2	97	53.5	0.001
3 or more	94	41.9	10	22.8	84	46.5	
Term of contract with employer (months)^b, c^							
1 - 2	25	11.1	8	18.5	17	9.3	0.393
3 - 6	73	32.7	12	28.4	61	33.8	
7 - 24	28	12.3	4	9.5	23	13.0	
Indefinite	44	19.4	6	13.1	38	21.0	
No written contract	55	24.4	13	30.5	41	23.0	
Ever had a serious accident at work that forced you to see a doctor^b^							
No	247	88.2	44	78.7	203	90.6	0.020
Yes	33	11.8	12	21.3	21	9.4	

Note 1: Totals may differ from the sum of categories due to rounding, unless specified

Note 2: Percentages are calculated based on the exact estimated standardized weighted counts

n, rounded estimated standardized weighted count

^a^, total percentage may differ from 100% due to rounding

^b^, total for the category may be less than the total n due to nonresponse

^c^, among the total full time and part time working population (n = 225)

The prevalence of health uninsurance was 19.8%. Those uninsured were more likely to be men (*P *= 0.006), have no children (*P *= 0.045), have less years of formal education (*P *= 0.013), do not know that health insurance was mandatory in Japan (*P *< 0.001), not have visited a doctor in the last 12 months (*P *< 0.001), not have a chronic disease for which they need to visit the doctor's office regularly (*P *= 0.016), have less years employed by the current employer (*P *= 0.001) and ever had a serious accident at work that forced them to see a doctor (*P *= 0.020).

The majority of respondents (73.3%) were employed through a labor contract company, but it was not associated with the insurance status. Those employed full time worked 9.86 (SE 0.18; median, 10.00) hours per day and 5.37 (SE 0.04; median, 5.00) days per week. On average, immigrants had been working for their current employers for much longer periods of time compared to the term of their contracts. Those working full time reported a term contract of 5.97 (SE 0.45; median, 6.00) months, but 3.23 (SE 0.35; median, 2.00) years working for the same employer. Shortest term of contract was 1-2 months and only observed among full time workers (data not shown in the tables). Sources of health insurance coverage were diverse but mostly from the National Health Insurance (NHI), irrespective of whether they were employed or not (Table 2). We also found that 6.0% of the insured had a health insurance provided by their labor contract company.

**Table 2:** Sources of health insurance by working condition among employed documented Latin American immigrants in Nagahama City, Japan

	National Health Insurance (NHI)	Employee's Health Insurance (EHI)	Labor contract company health insurance	Other public source	*P *value
	(n = 139)	(n = 70)	(n = 14)	(n = 3)	
	%	%	%	%	
Employed full time	55.5	35.9	8.1	0.5	0.007
Employed part time	72.7	27.3	0.0	0.0	
Unemployed	85.3	11.2	0.0	3.5	
Self employed/Retired	44.3	33.3	0.0	22.4	
Total	61.7	30.8	6.0	1.4	

n, rounded estimated standardized weighted count

Most uninsured respondents (68.3%) reported having ever had health insurance at some point in Japan, 64.6% had NHI and 47.4% had Employees' Health Insurance (EHI). However, only 37.1% think they will enroll in a health insurance plan in the next 6 months (data not shown in the tables). The reasons reported as the most important for lacking health insurance coverage are displayed in Table 3. Most respondents cited financial trade-offs as reasons for uninsurance. Many considered the health insurance too expensive (24.0%). In 12.2% of the cases, the respondents stated that the back payments for the time spent without being enrolled is too expensive and in 10.9% respondents said they would be leaving Japan soon. Employer's refusal of EHI was reported by only 2.0%.

**Table 3:** Most important reason for lack of health insurance among uninsured documented Latin Americans in Nagahama City, Japan

Reason	%
	(n = 56)
It's too expensive	24.0
I have too many years without insurance, it's too expensive to enroll now	12.2
I will be leaving Japan soon	10.9
I don't get sick so frequently	9.8
I have to save/send money	8.8
I don't have enough money to pay the health insurance	6.9
I or my spouse lost the job	6.1
Don't understand the Japanese insurance system	6.0
I changed employer	4.6
It's cheaper if I pay the medical expenses	4.0
In transition from another city	2.2
My employer refuses to enroll me in the Employees' Health Insurance (EHI)	2.0
Never thought about health insurance	1.3
If I get seriously sick/injured, I will return to my country	1.2

n, rounded estimated standardized weighted count

Regarding the correlates of uninsurance (Table 4), multiple logistic regression analysis showed that not having a chronic disease was the strongest predictor of uninsurance (AOR = 12.10). Also, those who do not know that health insurance is mandatory in Japan (AOR = 6.36), have one or no children (AOR = 2.58, 5.23), or have less education (AOR = 3.72) were significantly more likely to be uninsured.

**Table 4:** Correlates of uninsurance by multiple logistic regression analysis among documented Latin American immigrants in Nagahama City, Japan

	%	AOR	95% CI	*P *value
**Sociodemographic**				
Gender				
Men	55.6	2.63	(0.68 - 10.12)	0.147
Women	44.4	1.00		
Country of origin				
Bolivia	6.3	2.75	(0.47 - 16.16)	0.214
Others	19.9	0.25	(0.03 - 1.80)	
Brazil	73.8	1.00		
Number of children				
0	26.9	5.23	(2.132 - 12.89)	0.001
1	29.6	2.58	(0.50 - 13.21)	
2 or more	43.5	1.00		
Years of education				
1 - 8	14.1	3.72	(1.14 - 12.15)	0.032
9 or more	85.9	1.00		
**Knowledge about mandatory nature of health insurance in Japan**				
No	19.8	6.36	(1.34 - 30.25)	0.023
Yes	80.2	1.00		
**Medical background**				
Visit a doctor in Japan in the last 12 months				
No/Have never visited a doctor in Japan	31.3	2.72	(0.98 - 7.59)	0.055
Yes	68.7	1.00		
Chronic disease				
No	86.0	12.10	(1.56 - 94.09)	0.020
Yes	14.0	1.00		
**Work related**				
Years employed by current employer				
0 - 2	58.1	1.58	(0.43 - 5.85)	0.464
3 or more	41.9	1.00		
Ever had a serious accident at work that forced to see a doctor				
Yes	10.7	1.93	(0.68 - 5.47)	0.196
No	89.3	1.00		

AOR, Adjusted odds ratio

CI, Confidence interval

## Discussion

To our knowledge, this is the first study using rigorous methodology that demonstrates the pattern of health insurance coverage and the predictors of uninsurance among documented Latin American immigrants in Japan. We found that documented Latin American immigrants in Japan are uninsured at rates that are much higher than has been estimated in the general population (1.3%) [1], we found that almost 20% of the documented Latin American immigrants over 18 years of age lacked health insurance.

Among the immigrants themselves, the most common reason for uninsurance was considering the premiums too expensive. For workers who had not paid into the NHI for many years, back payments of premiums for the time they had lived in Japan without enrolling (up to a maximum of 2 years) to join/rejoin the NHI was also considered to be prohibitively expensive by many of the respondents. Low perceived medical needs and the expectation of a short-term stay in Japan were also among major reasons; these may reflect financial trade-offs. Some of the correlates of uninsurance from multivariate analysis are supportive in this respect since factors related to lower perceived medical needs such as not having or having fewer children, and not having a chronic disease were all strong predictors of uninsurance. Unwillingness of some immigrants to enroll in medical insurance is clearly represented by the fact that only one third of uninsured reported that they will enroll in a health insurance plan in the next 6 months.

In addition, we found that the majority of insured full time employees are covered by the National Health Insurance (NHI) rather than the Employees' Health Insurance (EHI). According to Japan's Health Insurance Act, employers are obligated to enroll their employees and their dependants in the EHI (except those employed 2 months or less, who work less than three quarters of the hours that full time employees work, and those aged 75 years or older) [29]. All those not eligible for EHI such as the self employed, unemployed, and retired younger than 75 years are covered by the NHI; and people aged 75 years and above are covered by the Late Elders' Health Insurance [30,31]. Health insurance coverage patterns revealed in our participants clearly deviate from the pattern required by the law. This could be discussed from two point of views.

First, immigrants' financial trade-offs may be responsible for the disproportionately low EHI coverage among employed immigrants as workers may select the NHI rather than the EHI; the latter mandatorily includes a pension premium, a preference that has been reported by other authors [14,32,33]. To receive a Japanese pension it is necessary to have paid into the system for 25 years and those who contributed fewer years than the stipulated, receive a maximum refund of only up to 3 years. Considering the fact that the majority of immigrants do not have intentions to stay permanently in Japan (in spite of the fact that most stay for many years), they may not have a strong incentive to enroll in the EHI.

Second, companies may be in part responsible for this inadequate and unusual coverage pattern. While the total employment period was about 3 years, the average contract period of full time workers was only 6 months. Furthermore, a high percentage of immigrants were working without a written contract. These mechanisms may allow the employer to avoid the obligation of providing EHI, and thus sparing half of the premium for the health insurance and the pension that is coupled with the EHI. Not providing EHI to full time employees who work longer than 2 months is illegal. This situation should be explored in further studies.

The high uninsurance rate among Latin American legal immigrants in Japan is troubling because it could have serious economic and health implications not only for workers themselves but also for their families. Immigrant workers have higher risks of occupational accidents and disability than native workers [34]. In our study uninsured respondents were even more likely to have had a serious accident at work than those insured. Occupational accidents may have resulted in financial burden which in turn lead to the choice of uninsurance, or employers may have been responsible for both, not providing working safety and health insurance. Whichever the case, outreach programs to persuade the immigrant workers about the importance of health insurance could be promoted through multiple partnerships among peer workers, Japanese care providers, researchers, and community leaders to help them adapt to a new health culture, and increase awareness about health. Such outreach has been used successfully to increase access to healthcare and health information in a culturally sensitive way in other countries [35-37]. Also, audit and legal enforcement on employers should be enhanced so that their employees are adequately insured.

To encourage immigrant employees to enroll in the EHI, policymakers in Japan should consider decoupling the pension premium from the health insurance premium. This would allow them to enroll in health insurance with a lower premium than the NHI. Alternatively, consideration could be given to promote a bilateral social security agreement between Japan and the country of origin of the immigrants to make the pension contributions effective in either country. Brazil is currently the only Latin American country to have recently signed an international social security agreement with Japan, though it is still awaiting implementation [38]. Of course, strict enforcement of the law is necessary to not allow the companies, especially labor contract companies through which the majority of immigrant workers are employed, escape the responsibility to cover their employees by the EHI. Foreign immigrant workers are placed into an unfair situation where the companies that indirectly employ immigrant workers through labor contract companies place responsibility on the labor contract companies, while labor contract companies may try to evade their responsibility to provide health insurance for their employees.

Finally, it should be noted that we found that some labor contract companies provided to their employees private health insurance plans. These programs should be closely monitored as they may allow companies to avoid their share of the premiums for the EHI and pension. Furthermore, health insurance provided by labor contract companies are not connected to the public health insurance system, thus immigrants may face back payments of premiums when they change employer and wish to join public health insurance programs.

Our findings should be interpreted in the context of their limitations. First, we need to consider the non coverage error [39] that may have arisen from the failure to include some immigrants from the selected clusters into the sampling framework during the mapping stage. Thus, the prevalence of uninsurance we found could in fact underestimate or overestimate the real value. Second, our findings may not be generalizable to other immigrant groups in Japan. However, we believe that our results shed light on the important fact that even in developed countries with universal health coverage, certain minority groups might be left out. Countries trying to achieve universal health coverage need to consider the vulnerability of these populations when planning and implementing reforms. Finally, as with any self reported data, the potential for reporting bias should be considered, as we were unable to verify their health insurance coverage or their legal status through other sources.

## Conclusions

We found that among our sample of legal Latin American immigrants in Japan, the proportion of people without health insurance was disproportionally high compared to the estimated national proportion. In addition, the coverage of employees by the EHI was disproportionally low. Appropriate measures should be taken to facilitate access to health insurance for this vulnerable population.

## Abbreviations

NHI: National Health Insurance; EHI: Employees' Health Insurance.

## Competing interests

The authors declare that they have no competing interests.

## Authors' contributions

All authors conceived and contributed to the study design. SPS conducted the fieldwork and MK supervised the data collection. SPS and MK carried out data analyses. SPS, MDF, and MK interpreted the data. SPS, MDF and MK drafted and revised the manuscript. MOK participated in the revision of the manuscript. All authors read and approved the final manuscript.

## Pre-publication history

The pre-publication history for this paper can be accessed here:

http://www.biomedcentral.com/1471-2458/12/238/prepub

## Supplementary Material

Additional file 1 **Table S1**. Additional information of documented Latin American immigrants in Nagahama City, Japan by health insurance status. Click here for file
