# Supplementary material for: Latin American immigrants have limited access to health insurance in Japan: a cross sectional study
Source: BMC Public Health. 2012 Mar 25;12:238. doi: 10.1186/1471-2458-12-238 (PMC3373364; doi:10.1186/1471-2458-12-238)
Supplement: Additional file 1 — Table S1. Additional information of documented Latin American immigrants in Nagahama City, Japan by health insurance status. [file 1471-2458-12-238-S1.DOC]

| **Table A1. Additional information of documented Latin American immigrants in Nagahama City, Japan by health insurance status.** | | | | | | | | | |
| --- | --- | --- | --- | --- | --- | --- | --- | --- | --- |
|  |  |  | Total | | Uninsured | | Insured | | *P* value |
|  |  |  | (n=282) | | (n=56) | | (n=226) | |
|  |  |  | n | %^a^ | n | %^a^ | n | %^a^ |
| **Sociodemographic** | | |  |  |  |  |  |  |  |
| Type of visa^b^ | | |  |  |  |  |  |  |  |
|  | Long term resident | | 156 | 55.6 | 38 | 68.4 | 118 | 52.5 | 0.416 |
|  | Permanent resident | | 94 | 33.4 | 15 | 26.3 | 79 | 35.1 |  |
|  | Other | | 31 | 11.0 | 3 | 5.3 | 28 | 12.4 |  |
| Years living in: | | |  |  |  |  |  |  |  |
|  | Japan^b^ | |  |  |  |  |  |  |  |
|  |  | Under 5 | 51 | 18.2 | 12 | 21.7 | 39 | 17.4 | 0.478 |
|  |  | 5 - 9 | 104 | 37.2 | 25 | 44.5 | 80 | 35.4 |  |
|  |  | 10 or more | 125 | 44.6 | 19 | 33.8 | 106 | 47.3 |  |
|  | Nagahama City | |  |  |  |  |  |  |  |
|  |  | Under 5 | 128 | 45.3 | 34 | 61.1 | 93 | 41.4 | 0.169 |
|  |  | 5 - 9 | 85 | 30.3 | 13 | 23.2 | 72 | 32.0 |  |
|  |  | 10 or more | 69 | 24.4 | 9 | 15.7 | 60 | 26.6 |  |
| **Intention to stay permanently in Japan?** | | |  |  |  |  |  |  |  |
|  | No / don't know | | 199 | 70.8 | 39 | 69.4 | 161 | 71.1 | 0.846 |
|  | Yes | | 82 | 29.2 | 17 | 30.6 | 65 | 28.9 |  |
| **Medical background** | | |  |  |  |  |  |  |  |
| Health insurance in the home country | | | |  |  |  |  |  |  |
|  | No | | 132 | 46.8 | 28 | 49.9 | 104 | 46.0 | 0.623 |
|  | Yes | | 150 | 53.2 | 28 | 50.1 | 122 | 54.0 |  |
| Ever been hospitalized in Japan^b^ | | |  |  |  |  |  |  |  |
|  | No | | 190 | 67.7 | 45 | 79.9 | 145 | 64.7 | 0.116 |
|  | Yes | | 90 | 32.3 | 11 | 20.1 | 79 | 35.3 |  |
| Ever felt so sick that needed to take days off from work^b^ | | |  |  |  |  |  |  |  |
|  | No | | 147 | 52.3 | 28 | 49.3 | 119 | 53.1 | 0.693 |
|  | Yes | | 134 | 47.7 | 28 | 50.7 | 105 | 46.9 |  |
| Family or friends ever been hospitalized in Japan^b^ | | |  |  |  |  |  |  |  |
|  | No | | 138 | 49.2 | 28 | 49.5 | 110 | 49.1 | 0.966 |
|  | Yes | | 142 | 50.8 | 28 | 50.5 | 114 | 50.9 |  |
| Note 1: Totals may differ from the sum of categories due to rounding, unless specified | | | | | | | |  |  |
| Note 2: Percentages are calculated based on the exact estimated standarized weighted counts | | | | | | | | |  |
| n, rounded estimated standardized weighted count | | | | |  |  |  |  |  |
| ^a^, total percentage may differ from 100% due to rounding | | |  |  |  |  |  |  |  |
| ^b^, total for the category may be less than the total n due to nonresponse | | | |  |  |  |  |  |  |
